# Auditory Dysfunction in Animal Models of Autism Spectrum Disorder

**DOI:** 10.3389/fnmol.2022.845155

**Published:** 2022-04-13

**Authors:** Ana Carolina Castro, Patricia Monteiro

**Affiliations:** ^1^Life and Health Sciences Research Institute, School of Medicine, University of Minho, Braga, Portugal; ^2^ICVS/3B’s–PT Government Associate Laboratory, Braga, Portugal

**Keywords:** autism spectrum disorder (ASD), sensory perception, sound processing, rodent models, auditory dysfunction

## Abstract

Autism spectrum disorder (ASD) is a neurodevelopmental disorder mainly characterized by social-communication impairments, repetitive behaviors and altered sensory perception. Auditory hypersensitivity is the most common sensory-perceptual abnormality in ASD, however, its underlying neurobiological mechanisms remain elusive. Consistently with reports in ASD patients, animal models for ASD present sensory-perception alterations, including auditory processing impairments. Here we review the current knowledge regarding auditory dysfunction in rodent models of ASD, exploring both shared and distinct features among them, mechanistic and molecular underpinnings, and potential therapeutic approaches. Overall, auditory dysfunction in ASD models seems to arise from impaired central processing. Depending on the model, impairments may arise at different steps along the auditory pathway, from auditory brainstem up to the auditory cortex. Common defects found across models encompass atypical tonotopicity in different regions of the auditory pathway, temporal and spectral processing impairments and histological differences. Imbalance between excitation and inhibition (E/I imbalance) is one of the most well-supported mechanisms explaining the auditory phenotype in the ASD models studied so far and seems to be linked to alterations in GABAergic signaling. Such E/I imbalance may have a large impact on the development of the auditory pathway, influencing the establishment of connections responsible for normal sound processing.

## Introduction

Autism Spectrum Disorder (ASD) is a neurodevelopmental disorder with a poorly understood etiology. A recent whole-exome sequencing study identified 102 candidate genes mainly responsible for regulation of gene expression and synaptic neuronal communication (Satterstrom et al., 2020). But besides its clear genetic origins, some environmental factors can increase the risk of ASD, especially during critical periods of embryonic development (Arndt et al., 2005). In accordance, most candidate genes from the whole-exome sequencing study are expressed exactly during development and display cortical enrichment in maturing excitatory and inhibitory neuronal lineages.

Regarding clinical manifestations, ASD is characterized by deficits in social communication and interaction, and repetitive patterned behaviors or restricted interests (American Psychiatric Association [APA], 2013). Recently, abnormal sensory sensitivity was also included in ASD diagnosis. This comprises hyper- or hyposensitivity to sensory inputs from vision, audition, touch, smell and taste, or unusual interest in sensory aspects of the environment (American Psychiatric Association [APA], 2013; Robertson and Baron-Cohen, 2017). These sensory-perceptual abnormalities are present in approximately 90% of individuals (Leekam et al., 2007; Tomchek and Dunn, 2007; Crane et al., 2009), being auditory hypersensitivity the most common sensory-perceptual abnormality (Gomes et al., 2008). By perceiving auditory inputs as noxious or unpleasant, patients may instinctively learn to avoid them (Marco et al., 2011), which could potentially be the root for the communication, socialization and learning impairments observed in ASD. Given the pertinence of understanding how these auditory-perceptual alterations may contribute to ASD, animal models are crucial tools not only to study its neurobiological underpinnings but also to dissect potentially shared mechanisms across different ASD models from multiple origins.

**FIGURE 1:**
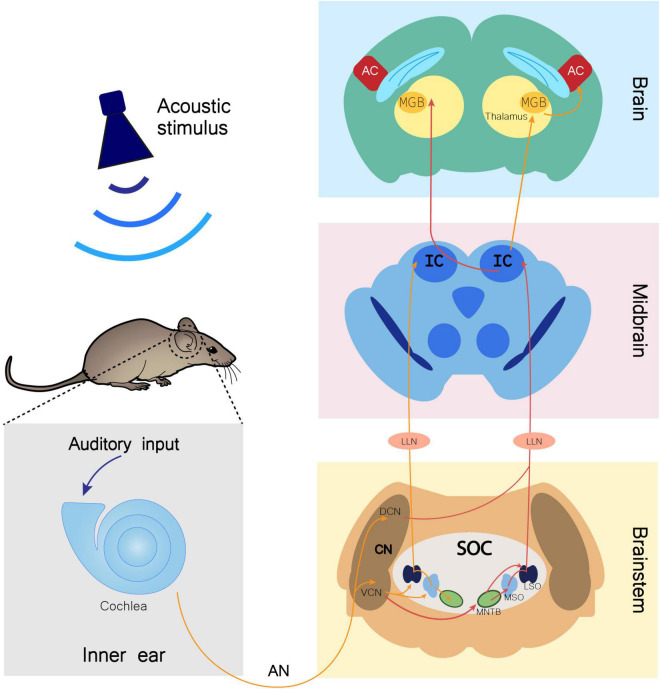
Monoaural ascending auditory pathway. Auditory input arriving to the cochlea is transmitted through the auditory nerve (AN) to the cochlear nucleus (CN) in the brainstem. In this nucleus, AN bifurcates to the ventral cochlear nucleus (VCN) and the dorsal cochlear nucleus (DCN). Information is directed to the ipsilateral (orange arrows) and contralateral (red arrows) SOC (superior olivary complex), traveling through several of its nuclei, namely the lateral superior olive (LSO), medial superior olive (MSO), and medial nucleus of the trapezoid body (MNTB). Both contralateral and ipsilateral input from the brainstem reach the inferior colliculus (IC) in the midbrain through fibers of the lateral lemniscus (LL), synapsing in the LL nucleus (LLN). From the IC, fibers project ipsilaterally and contralaterally to the medial geniculate body (MGB). The MGB is located in the thalamus and is the brain region that precedes the auditory cortex (AC) in the information flow of ascending auditory pathway (Knipper et al., 2013; Di Bonito and Studer, 2017).

Upon an auditory stimulus, the nervous impulse travels through the auditory nerve (AN) until a relay center in the brainstem, the cochlear nucleus (CN), which is mainly divided in dorsal (DCN) and ventral (VCN) regions. The superior olivary complex (SOC) receives input from the CN and has three nuclei involved in auditory input processing: the lateral superior olive (LSO), the medial superior olive (MSO), and the medial nucleus of the trapezoid body (MNTB). The SOC then projects to the inferior colliculus (IC) through the fibers of the lateral lemniscus (LL), synapsing in the LL nucleus (LLN). From the IC, information travels to the medial geniculate body (MGB), which lies in the thalamus and is the last auditory center before reaching the auditory cortex (AC), conveying information from several regions of the auditory system (Figure 1; Malmierca, 2003; Knipper et al., 2013; Di Bonito and Studer, 2017).

**FIGURE 2:**
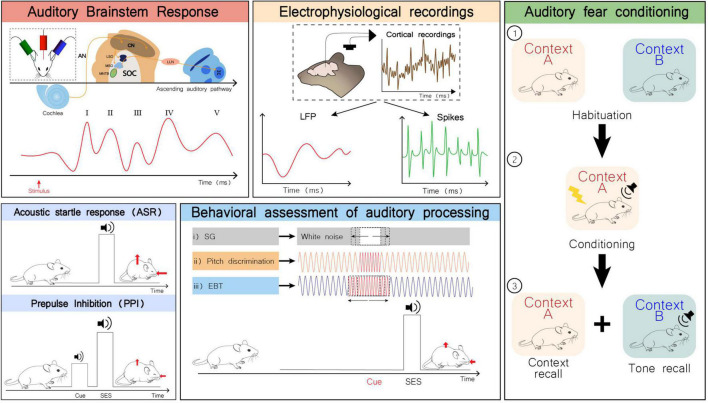
Examples of some experimental approaches to study auditory alterations in rodents. Auditory brainstem response (ABR): Schematic representation of electrodes placement for ABR recording (inset; top left). Representation of the auditory pathway and corresponding ABR waves generated upon sound stimulation. Measuring ABR is a non-invasive to assess the sum of evoked potentials that occur in the first milliseconds after sound stimulation across the auditory system (Moller, 2006). Wave I is generated by signal propagation through the auditory nerve (AN), wave II through the cochlear nuclei (CN), wave III through the superior olivary complex (SOC), wave IV through the lateral lemniscus (LL), and wave V through the inferior colliculus (IC) (Knipper et al., 2013). LSO, lateral superior olive; MSO, medial superior olive; MNTB, medial nucleus of the trapezoid body. Electrophysiological recordings: Local field potentials (LFP) and spiking activity can be obtained from *in vivo* electrophysiological recordings in the auditory cortex. LFPs represent the summed activity of a certain population of neurons that is recorded in a specific region, while spiking refers to a single neuron activity. Acoustic startle response (ASR) and prepulse inhibition (PPI): The ASR is a short-latency response to a startle eliciting stimulus (SES), considered a defensive reaction to a strong and sudden stimulus that prepares the individual to face a potential threat. It is characterized by a rapid contraction of facial and skeletal muscles (red arrows). This response is preserved across mammalian species, involving the brainstem, and the auditory and vestibular systems. The PPI test evaluates the attenuation of the ASR by presentation of a non-startling stimulus immediately preceding the SES. This enables to quantitatively assess the sensory-motor gating function, and the ability to effectively filter sensory information, hence preventing sensory overload (Koch and Schnitzler, 1997; Gómez-Nieto et al., 2020). Behavioral assessment of auditory processing: representation of behavioral paradigms to measure the ASR attenuation by a previously presented auditory cue. While in PPI test the type of stimulus used as cue is the same as in the SES, this set of behavioral tests takes advantage of other types of auditory cues for ASR inhibition. Adequately processing these auditory cues underlies an effective ASR inhibition, which in turn becomes a measure for auditory discrimination of different types of auditory cues. (i) The subject is exposed to a white-noise background and a silence gap of variable duration in the background noise is used as cue for the upcoming SES; (ii) and (iii) The subject is exposed to a background pure tone. The cue preceding the SES is a momentaneous change in frequency. In (ii) the duration of the cue is constant, but the frequency varies between trials and in (iii) the duration of the cue is variable, but its frequency is the same across trials (Truong et al., 2015; Gómez-Nieto et al., 2020). Fear conditioning paradigm: (1) Subjects are habituated to contexts A and B; (2) Subjects are conditioned in context A by pairing a tone with shock; (3) Context and tone recall are performed 24 h later. Freezing behavior is evaluated and compared across the whole test (Reinhard et al., 2019). Although the central role of the amygdala in the process of fear conditioning is well-accepted, this is not the only critical player in this process. The auditory thalamus is increasingly considered as an important sensory integration center in the network involved in fear conditioning. Hence, this paradigm may also provide valuable information regarding auditory processing (Gründemann, 2021).

Given the broad etiology of ASD, many animal models have been developed to uncover ASD’s molecular underpinnings. These models are based in genetic and non-genetic factors associated with increased risk for autism. Other models not specifically conceived to study ASD display some ASD-like behaviors and may be useful for clarifying potentially shared mechanisms. In this review, we will summarize the current knowledge regarding auditory alterations that have been found through multiple experimental approaches (Figure 2), as well as physiological, anatomical, and functional alterations identified in several rodent models of ASD (Supplementary Table 1).

### Non-genetic Models of Autism Spectrum Disorder

Some of the most relevant environmental risk factors for ASD are: parental age, maternal nutrition, diseases, and infections; fetal exposure to teratogenic drugs, alcohol or other toxic compounds; and complications during delivery (e.g., perinatal hypoxia) (Burd et al., 1999; Masini et al., 2020). In this review, we will summarize auditory processing deficits in animals that display ASD-like behaviors upon exposure to two environmental factors: valproic acid, and thalidomide.

####  Valproic Acid

Prenatal exposure to the antiemetic drug thalidomide, the anticonvulsant drug valproic acid (VPA), and the antidepressant drug citalopram, affect neurodevelopment and behavior (Arndt et al., 2005; Maciag et al., 2006). Rodents exposed to VPA *in utero* are perhaps the most studied environmental model of ASD, displaying impaired social interaction, abnormal sensory reactivity, anxiety-like, and repetitive behaviors (Gandal et al., 2010; Engineer et al., 2014a; Nagode et al., 2017).

Valproic acid-exposed mice show delayed ear opening (Zimmerman et al., 2018) and reduced ultrasonic vocalizations (USVs), both in early-life stages (decreased pup distress calls) and after sexual maturation (pre-mating vocalization) (Gandal et al., 2010). Their acoustic startle response (ASR) is normal but prepulse inhibition (PPI) is decreased, indicating impairments in sensory motor gating without hypo- or hyperreactivity to sound (Gandal et al., 2010).

Their primary and non-primary regions of the AC show alterations according to stimulus type (speech sound, noise burst, or pure tone) (Engineer et al., 2014a; Anomal et al., 2015). Weaker local field potentials (LFPs) in the anterior auditory field (AAF), but stronger in the primary auditory cortex (A1), are evoked by speech sounds. In response to pure tones, AAF presents the weakest, most delayed responses. Temporal and spectral processing is mainly impaired in the AAF, and this area seems to be the most affected regardless of stimulus type (Engineer et al., 2014a). Regarding A1, VPA-exposed rats exhibit a disorganized tonotopic map with overrepresented higher frequencies and receptive fields less accurately tuned to characteristic sound frequencies (CFs). Onset latency for neuronal firing in response to tones is decreased in A1 of VPA-exposed rats (Anomal et al., 2015). Although it has been suggested that a shorter inhibition in post-stimulus suppression may underlie these impairments (Engineer et al., 2014a), it is perhaps worth mentioning that number and density of parvalbumin-positive (PV^+^) neurons in A1 is not changed (Anomal et al., 2015).

Observations from ASD patients and VPA-exposed rodents both indicate hypoplasia and dysmorphology of the brainstem (Dubiel and Kulesza, 2016). After pure tone sound exposure, c-Fos immunolabeling of the brainstem auditory centers suggests a generalized overactivation (Dubiel and Kulesza, 2016). In the CN and MNTB of VPA-exposed animals it is noticeable an overactivation together with tonotopical disruption (neuronal activation is observed beyond its normal tonotopic bands) (Dubiel and Kulesza, 2016). Additionally, VPA exposure leads to the development of fewer, smaller, and irregularly shaped neurons across the entire SOC (Lukose et al., 2011; Zimmerman et al., 2018, 2020). This happens at the level of several nuclei of the auditory brainstem (CN, SOC, LLN) with special impact on MNTB (Dubiel and Kulesza, 2016; Zimmerman et al., 2018).

Prenatal VPA exposure also seems to induce deficits in the expression of calbindin (CB) in principal neurons and octopus cells of the MNTB, as well as deficits in the expression of calretinin (CR) in the globular bushy cells of the VCN (Zimmerman et al., 2018). This may destabilize calcium signaling on those cells, leading to problems in sound localization and temporal coding (Zimmerman et al., 2018).

Valproic acid-induced alterations in the brainstem seem to arise from developmental differences in synaptic maturation with decreased inhibition (GABAergic signaling) or increased excitation (glutamatergic signaling). Other possibility is an excitatory hyperconnectivity promoted by abnormal axonal projections (Dubiel and Kulesza, 2016; Zimmerman et al., 2018). Interestingly, VPA exposure seems to increase postsynaptic glutamatergic excitatory connections between cortical plate and subplate (L5/6 and L4) by the second postnatal week (Nagode et al., 2017). By the same period, there is an increase in GABAergic inhibitory connections to subplate neurons. Although such concomitant increase in excitatory and inhibitory connections could in principle reflect a preservation effect of the excitatory/inhibitory (E/I) balance, the effect of VPA over inhibition seems to be greater than over excitation, affecting final E/I balance in the developing A1 (Nagode et al., 2017).

Lastly, VPA model presents reduced proportion of neurons sending ascending projections from NLL, SOC, and CN to the central nucleus of the IC (CNIC). This impacts connectivity between brainstem and midbrain, hence, sound processing in the midbrain (Zimmerman et al., 2020), which seems to be particularly susceptible to VPA damage. The CNIC and NLL present reduced size, reduced neuronal density, and abnormal neuronal morphology. A reduction in CB^+^ neurons was also reported in the dorsal nucleus of the LL (DNLL) and reduced dopaminergic input to the CNIC can be assumed based on reduced TH^+^ (tyrosine hydroxylase positive) labeling in this region (Mansour et al., 2019). The CNIC projects to the MGB, that is also affected in VPA model, presenting fewer and smaller neurons and receiving less input from the midbrain and brainstem (Mansour et al., 2021).

#### Thalidomide

Many of the findings from the VPA model are also true for the thalidomide model, although fewer studies are available and focus essentially on MNTB alterations. MNTB is reported to be functionally impaired and diminished in size, with decreased projecting neuronal fibers, and increased c-Fos expression (expanded responsive area), upon tone stimulation (Ida-Eto et al., 2017; Tsugiyama et al., 2020). In line with the VPA model, decreased levels of CB expression are apparent in the SOC of thalidomide model (Tsugiyama et al., 2020).

### Monogenic Models of Autism Spectrum Disorder

Despite the apparently diverse genomic landscape behind ASD (Betancur, 2011), convergent molecular functions have been found for many ASD-candidate genes. Animal models are crucial tools to uncover such findings and besides gene function, important work has been carried out regarding auditory deficits.

#### *Fmr1* Knockout

Mutations in the *FMR1* gene are responsible for fragile X syndrome (FXS) and contribute to 8% of ASD cases (Zhang et al., 2019). The *FMR1* gene encodes the fragile X mental retardation protein (FMRP), which is an RNA-interacting protein that shuttles RNA from the nucleus to neuronal dendrites. FMRP seems to be a key player in dendritic spine formation (Braun and Segal, 2000), contributing to excitatory synapses and brain plasticity (Bassell and Warren, 2008; Sethna et al., 2014). *Fmr1*-KO are used as FXS models and typically display hyperactivity and learning deficits (The Dutch-Belgian Fragile X Consorthium et al., 1994).

When exposed to high intensity sounds, *Fmr1*-KO mice present increased tendency to audiogenic seizures that increases with age (Chen and Toth, 2001). According to the same study, these mice also present lower ASR and more efficient PPI. However, ASR was tested for one sound intensity only (no startle response curve reported), hence information on ASR threshold is absent and could potentially be an indicator of auditory sensitivity (Chen and Toth, 2001).

Regarding auditory fear conditioning, *Fmr1*-KO mice show lower freezing response to conditioned-unconditioned paired stimulus and to tone recalling (Reinhard et al., 2019). Such reduced freezing does not seem to be due to genotype-associated differences in locomotion but rather due to sound processing impairments in the KO (Reinhard et al., 2019). Reinforcing this, it was observed that *Fmr1*-KO rats present temporal integration of sound stimulus impaired but enhanced spectral integration. Since they present faster reaction times in response to a wider range of sound intensities, there is evidence of sound hyperacusis (Auerbach et al., 2021). Lack of FMRP does not affect the ability to discriminate phonemes, suggesting that speech sound discrimination is likely normal (Engineer et al., 2014b).

Further evidence for the presence of auditory deficits in the *Fmr1* model has been obtained from electrophysiological studies where functional cortical impairments have been found (Rotschafer and Razak, 2013; Engineer et al., 2014b). KO cortical neurons show normal intensity threshold but enhanced response magnitude to tones, as well as broader frequency tuning. Such enhanced magnitude might arise from an inability to shut down neuronal activity during sound exposure (Rotschafer and Razak, 2013). When KO mice are exposed to trains of noise, the obtained responses differ between the first and subsequent stimulus. Initially, weaker responses are recorded at the AAF and ventral auditory field (VAF), but after several trains of noise, VAF presents weaker response, and the posterior auditory field (PAF) becomes stronger. The overall most affected area seems to be VAF (Engineer et al., 2014b). *Fmr1*-KO also present less selectivity to frequency modulated (FM) sweep rates and higher response to fast sweep rates, without differences regarding direction selectivity (up- or downward sweeps) (Rotschafer and Razak, 2013). Regarding AC response to speech sounds, evoked LFPs display lower peak amplitudes (N1, P2, N2, and P3 components) in *Fmr1*-KO. When splitting AC data into four fields (AAF, A1, VAF, and PAF), A1 and VAF show the weakest amplitudes for all components (Engineer et al., 2014b). Furthermore, onset latency in PAF and VAF is increased but temporal precision and strength to speech sounds are reduced (Engineer et al., 2014b).

*Fmr1*-KO mice are reported to have higher ABR thresholds for click sounds and pure tone frequencies, and reduced peak I and III amplitudes, suggesting defects in AN and brainstem. Response latency to sound stimulation and inter-peak latency are not altered (Rotschafer et al., 2015).

Histological assessments in the auditory brainstem of adult *Fmr1*-KO mice further reveal that neuronal cell size is reduced in VCN, MSO, and MNTB (not in LSO) (Rotschafer et al., 2015; Ruby et al., 2015). Interestingly, although several cellular defects can be detected since birth at MNTB, these only emerge at VCN after hearing onset (Ruby et al., 2015; Rotschafer and Cramer, 2017). Of note, FMRP is expressed across the auditory brainstem in a specific pattern. In MNTB it has a tonotopical expression similar to potassium channel Kv3.1b, a channel that seems crucial for normal sound processing specifically in the binaural sound localization circuit (Strumbos et al., 2010; Ruby et al., 2015). In *Fmr1*-KO, however, the typical expression pattern of Kv3.1b in MNTB is not observed, namely its tonotopical gradient or increased expression upon sound stimulation (Strumbos et al., 2010; Ruby et al., 2015). Additionally, *Fmr1*-KO mice present reduced axonal profiles and CB immunoreactive terminals in MNTB somata which may indicate abnormal calcium signaling regulation (Ruby et al., 2015).

Focusing on two important auditory nuclei of the SOC (MNTB and LSO), one study found a greater strengthening of excitatory input from the VCN to the LSO in *Fmr1*-KO, likely due to an increased number of synaptic connections (Garcia-Pino et al., 2017). Besides receiving excitatory input from the VCN, the LSO receives inhibitory input from the MNTB, which seems unaffected in *Fmr1*-KO mice. Therefore, the net effect is a potential increase in excitation. Such changes in E/I balance could explain the increased firing rates and broadened tuning curves observed in the LSO of *Fmr1*-KO mice and might contribute to their reported acoustic hypersensitivity (Garcia-Pino et al., 2017).

Auditory stimulation with tone bursts and amplitude-modulated tones leads to increased activation of IC in *Fmr1*-KO mice, especially in neurons that respond to lower frequencies (<20 kHz). Broader tuning frequency is generally observed in individual neurons (Nguyen et al., 2020). Although the development of connections within the auditory circuitry seems to occur normally in *Fmr1*-KO, sound-driven refinements of excitatory inputs seem affected by the time of hearing onset (∼P10-P11) (Nguyen et al., 2020). Because these refinements depend on precisely timed inhibitory inputs, it is thought that impairments in GABAergic signaling may cause E/I defects in IC. Of note, neurons in the dorsal region of the IC present a CF < 20 kHz, being the most responsive in *Fmr1*-KO and more hypersensitive. Given the prominent role of GABA in the dorsal region of the IC, it seems that GABAergic dysfunction may underlie abnormal responsiveness in this area (Nguyen et al., 2020).

In a broader picture, there is evidence consistent with both an increase in excitation mediated by strengthening of excitatory inputs and decrease in inhibition due to impairments on GABA-related neurotransmission. Thus, the E/I imbalance often described as a hallmark of ASD seems to hold true in the *Fmr1*-KO model. At the molecular level, one of the possible candidates underlying this imbalance and contributing to auditory hypersensitivity in *Fmr1*-KO is matrix metalloproteinase-9 (MMP-9). This enzyme is upregulated in the AC of *Fmr1*-KO mice and has been associated with its reduced ERP habituation (event related potentials) (Lovelace et al., 2016, 2018, 2020b). Furthermore, MMP-9 upregulation likely hinders the formation of perineuronal nets (PNNs) around parvalbumin-expressing (PV^+^) cortical interneurons (GABAergic neurons), leading to reduced cortical network inhibition and E/I imbalance in the AC (Wen et al., 2018). Genetic or pharmacological strategies for reducing MMP-9 levels seem to rescue the following phenotypes: (1) audiogenic seizure susceptibility, (2) AC activity (spontaneous and evoked), (3) auditory ERP habituation, (4) formation of PNNs around PV^+^ cells, (5) anxiety-like and hyperactive behaviors, (6) ASR (Gkogkas et al., 2014; Lovelace et al., 2016, 2020a; Wen et al., 2018; Kokash et al., 2019; Pirbhoy et al., 2020). Enhancement of endocannabinoid production (2-arachidonoyl-sn-glycerol) seems to ameliorate synchrony in the cortical response to auditory stimuli, and rescue anxiety-like and hyperactive behaviors (Pirbhoy et al., 2021). Inhibiting phosphodiesterase 10A seems an additional therapeutic approach since it was shown to improve auditory processing in *Fmr1*-KO mice (Jonak et al., 2021). Interestingly, sound exposure during postnatal development seems to rescue/normalize ERP, PV^+^ cell density and dendritic spine density in the AC (Kulinich et al., 2020).

#### *Shank3* Knockout

The *SHANK3* gene encodes a scaffold protein (SHANK3) enriched in the post-synaptic density of excitatory synapses and is currently one of the best characterized risk genes in ASD (Monteiro and Feng, 2017). Several studies have shown that *Shank3* mutations in zebrafish (Liu et al., 2018), mice (Mei et al., 2016; Zhou et al., 2016), rats (Song et al., 2019), and even primates (Zhou Y. et al., 2019), lead to reduced social interaction and repetitive behaviors, as well as other numerous defects (Peça et al., 2011; Zhou et al., 2016; Delling and Boeckers, 2021).

Being *SHANK3* haploinsufficiency a clear monogenic cause of ASD, dozens of mouse lines carrying different *Shank3* mutations have been generated so far (Monteiro and Feng, 2017). One in particular, carries a human genetic mutation from an ASD-patient—the *InsG3680-Shank3* mouse line. This model displays increased ASR, which indicates a possible impairment in sound processing and potential auditory hypersensitivity. PPI seems to be decreased in this model, although the baseline acoustic reactivity may portrait a confounding factor to this test, as discussed by Zhou et al. (2016). In a different model, the *Shank3B*-KO, no differences were found in PPI or silent gap (SG) test (Rendall et al., 2019). On the other hand, *Shank3B*-KO seem to have increased ability to discriminate pitch changes (Rendall et al., 2019).

Impairments in cortical sound processing are reported for heterozygous *Shank3*-deficient rats carrying a 68 bp deletion in exon six with a premature stop codon (Engineer et al., 2018). Cortical response to tone stimulus, noise bursts, and speech sounds, are overall weaker in these rats, but temporal properties seem to be unaltered in response to tones. The same is not true for response to speech sounds, that besides being weaker, is also delayed. Of note, although neural discrimination accuracy does not seem to be impaired when sounds are presented in an isolated manner, neural responses are degraded when sounds are presented at increased rates, such as human speech rate (Engineer et al., 2018).

#### α7-nAChR Knockout

*CHRNA7* is a gene encoding for α7-nicotinic acetylcholine receptor (α7-nAChR), an homopentameric transmembranar protein highly expressed in the brain (Schaaf, 2014). α7-nAChR expression starts during prenatal development and peaks during the first synaptogenesis events. Changes in channel expression negatively influence neurogenesis, synaptogenesis and neuroblasts’ migratory events (De Jaco et al., 2016). In humans, microduplications in this gene are associated with a wide range of neurobehavioral disorders, including ASD (Dennis et al., 2012).

In rodents, α7-nAChR loss is associated with developmental impairments that affect auditory processing. ABR hearing thresholds and peak amplitudes are unaffected, but peak IV latency is increased, suggestive of impairments in the midbrain. Accordingly, single unit responses recorded in the IC revealed that KO animals have a subset of neurons with an atypical response to pure tones, presenting also deficits in spike timing, forward masking, and silent gap detection (Felix et al., 2019). In more detail, evoked responses in the IC are typically transient (1–40-ms duration) and sustained (80–120-ms). In the KOs, however, a third type of response can be detected with intermediate response (40–80-ms) (Felix et al., 2019). Degraded spike timing was also found in the ventral nucleus of the LL (VNLL) and superior paraolivary nucleus (SPON), which are primary targets of octopus cells (highly temporal precise cells likely responsible for shaping temporal responses in the midbrain) (Felix et al., 2019).

#### *Cntnap2* Knockout

Contactin-associated protein-like 2 (*CNTNAP2*) is an ASD-related gene implicated in language impairments (Rodenas-Cuadrado et al., 2014). The encoded protein is responsible for tethering potassium channels in myelinated axons, being crucial for action potential propagation (Truong et al., 2015). Loss of Cntnap2 in rodents leads to alterations in social behavior, reduced USVs, and hyperactivity (Peñagarikano et al., 2011; Brunner et al., 2015).

Conclusions regarding sensory-motor gating ability of *Cntnap2*-KO mice are not consensual. Different studies report either unchanged (Peñagarikano et al., 2011), impaired (Scott et al., 2018), or more efficient PPI (Brunner et al., 2015; Truong et al., 2015). The same is observed regarding ASR whose changes may impact PPI and explain results disparity (probably also age factor). A battery of tests based on PPI paradigm have been performed in *Cntnap2*-KO. By changing the type of prepulse cue, it is possible to detect impaired ability to perceive short SGs in a continuous broadband white noise background, but increased ability to discriminate slight changes in pitch (Truong et al., 2015). In order to elicit a startle inhibition with SGs, these must be much longer, suggesting that *Cntnap2*-KO have impairments in temporal sound processing. Ability to attenuate a startle response when a pitch cue was presented was, however, independent of pitch tone duration. The auditory alterations reported in this model seem to be linked to MGB neuronal changes (Truong et al., 2015).

Analysis of ABR to click sounds and pure tones in *Cntnap2*-KO rats shows altered peak amplitudes and latencies but overall unaffected hearing thresholds. In more detail, peaks II, III, and IV latencies were observed to be consistently increased in juvenile rats, although this trait was recovered in adulthood. The amplitude of peak IV was decreased across development and in adulthood. Interpeak latencies were also affected, mostly the latency between peaks I and II which was decreased both during development and adulthood (Scott et al., 2018).

Histological data shows reduced neuronal count and size in the MGB, which may help explain sound processing abnormalities. Given the results from ABR testing, further histological data would be useful to clarify the extent of changes in this model, similarly to other ASD models presented in this review (Truong et al., 2015; Scott et al., 2018).

#### *Pten* Conditional Knockout

*PTEN* mutations have been identified in individuals diagnosed with ASD and also displaying macrocephaly (Zhou and Parada, 2012). Accordingly, *PTEN*-KO mice present several alterations namely increased neuronal soma size, hypertrophic dendrites, higher excitatory spontaneous activity, and hypertrophic and ectopic dendrites (Xiong et al., 2012). Such alterations are also consistent with the known role of this gene, which is required for normal brain wiring and development.

*Pten*-KO pups tend to present increased frequency of USVs when separated from their mothers, a result interpreted as evidence of higher anxiety (Sarn et al., 2021). Conditional KO of *Pten* in the left AC of mice increases the strength of callosal inputs to that region and the efficiency of excitatory long-range synaptic inputs from contralateral AC and thalamus. An increase in dendritic branch number and spine density together with increased amplitude of miniature excitatory postsynaptic currents is consistent with overall strengthening of synaptic connections in this region (Xiong et al., 2012).

#### *Mecp2* Transgenic Mouse

The *MECP2* gene encodes methyl-CpG binding protein (MeCP2) that acts as a regulator of gene expression, playing an important role in prenatal neurogenesis and postnatal synaptic development, function, and plasticity (Brand et al., 2021). *MECP2* genetic loss of function is associated with intellectual disability and Rett syndrome (Brand et al., 2021), whereas its duplication is characterized by motor and cognitive impairments, delayed or absent speech, seizures, and ataxia (D’Mello, 2021). In both situations, ASD-related phenotypes are often times present.

Altered sound-evoked cortical responses have been reported in *Mecp2-*overexpressing transgenic mice (*Mecp2*-TG). Although displaying normal CF distribution, thresholds to trigger tone-evoked cortical responses are increased. In contrast, cortical responses to noise are stronger, but delayed. Such abnormalities indicate a noise sensitivity phenotype associated to fast-spiking neurons that might be due to lack of cortical inhibition (Zhou C. et al., 2019). Unlike many other ASD models, ABR is normal in *Mecp2* overexpressing mice and cortical tonotopy does not seem to be affected (Zhou C. et al., 2019).

### Other Autism Spectrum Disorder Models Displaying Auditory-Related Impairments

Although far less explored, there are other animal models that potentially display auditory processing abnormalities. The *Cyfip1*^±^ mouse model presents a lower PPI, evidencing sensory-motor gating impairments (Domínguez-Iturza et al., 2019) and the *Nrxn1*α-KO presents increased ASR without changes in PPI, perhaps due to acoustic hypersensitivity (Esclassan et al., 2015). However, PPI results in *Nrxn1*α-KO rats may be confounded by their decreased ASR (Esclassan et al., 2015). *Adnp*^±^ mice display increased ABR thresholds and latency, as well as decreased number of USV calls. Such findings might be a consequence of the reported significant hearing loss displayed by *Adnp*^±^ mice (Hacohen-Kleiman et al., 2019).

## Conclusion and Future Perspectives

Increasing evidence from animal models demonstrates that auditory-perceptual alterations found in ASD patients can be recapitulated in several animal models. Such neurodivergent processing of auditory inputs, translated into hypo- or hypersensitivity to sensory stimulation, may have a strong impact on behavior and impose limitations in the quality of life for individuals diagnosed with ASD. In particular, auditory processing abnormalities may underlie deficits in communication and social interaction. Tackling the neurobiological mechanisms causing such alterations becomes of utmost importance to design strategies to attenuate or prevent sensory impairments. Rodent models are a powerful resource to better understand behavioral and neurobiological alterations in ASD, holding tremendous translational potential.

Despite the great diversity of ASD models, mirroring the great heterogeneity in the etiology of ASD, it is possible to identify shared features across models. Auditory impairments seem to arise from deficits in central processing rather than from periphery. Along the auditory pathway, multiple defects are observed, such as decreased tonotopicity, altered thresholds to sound stimuli, and abnormal spectral and temporal processing (especially in the auditory regions of the brainstem and cortex). The origin of these differences is still not fully understood, but E/I imbalances during postnatal development seem to be contributing to these defects, mainly due to impairments in GABAergic signaling.

Future work is needed to better support these observations and unveil specific regions, neuronal circuits, and molecular players that are determinant for the auditory phenotype. Since ASD is a neurodevelopmental disorder, clarifications on critical developmental stages will be crucial, together with the establishment of novel molecular targets that might be particularly effective during those developmental windows. Together, this knowledge will hopefully help defining efficient therapeutic approaches in the near future.

## Author Contributions

AC conceptualized and wrote the manuscript. PM conceptualized and revised the manuscript. Both authors contributed to the article and approved the submitted version.

## Conflict of Interest

The authors declare that the research was conducted in the absence of any commercial or financial relationships that could be construed as a potential conflict of interest.

## Publisher’s Note

All claims expressed in this article are solely those of the authors and do not necessarily represent those of their affiliated organizations, or those of the publisher, the editors and the reviewers. Any product that may be evaluated in this article, or claim that may be made by its manufacturer, is not guaranteed or endorsed by the publisher.

## Figures and Tables

**Table:** 

A1	Primary auditory cortex	ERP	Event related potentials	PAF	Posterior auditory field
AAF	Anterior auditory field	FM	Frequency modulated	PNN	Perineuronal net
ABR	Auditory brainstem response	FMRP	Fragile X mental retardation protein	PPI	Prepulse inhibition
AC	Auditory cortex	FXS	Fragile X syndrome	PV	Parvalbumin
AN	Auditory nerve	IC	Inferior colliculus	SES	Startle eliciting stimulus
ASD	Autism Spectrum Disorder	LFP	Local field potential	SG	Silent gap
ASR	Acoustic startle response	LL	Lateral lemniscus	SOC	Superior olivary complex
CB	Calbindin	LLN	Lateral lemniscus nucleus	SPON	Superior paraolivary nucleus
CF	Characteristic sound frequency	LSO	Lateral superior olive	TH	Tyrosine hydroxylase
CN	Cochlear nucleus	MeCP2	Methyl-CpG binding protein	USV	Ultrasonic vocalization
CNIC	Central nucleus of the inferior colliculus	*Mecp2*-TG	*Mecp2*-overexpressing transgenic mice	VAF	Ventral auditory field
*CNTNAP2*	Contactin-associated protein-like 2	MGB	Medial geniculate body	VCN	Ventral cochlear nucleus
CR	Calretinin	MMP-9	Matrix metalloproteinase-9	VNLL	Ventral nucleus of the LL
DCN	Dorsal cochlear nucleus	MNTB	Medial nucleus of the trapezoid body	VPA	Valproic acid
DNLL	Dorsal nucleus of the lateral lemniscus	MSO	Medial superior olive	α7-nAChR	α7-nicotinic acetylcholine receptor
