# Supplementary material for: Auditory Dysfunction in Animal Models of Autism Spectrum Disorder
Source: Front Mol Neurosci. 2022 Apr 13;15:845155. doi: 10.3389/fnmol.2022.845155 (PMC9043325; doi:10.3389/fnmol.2022.845155)
Supplement: Supplementary file 1 [file Table_1.pdf]

Supplementary Table S1 – Overview of the most relevant findings regarding ASD rodent models with reported auditory dysfunctions. Abbreviations: acoustic startle response (ASR), prepulse inhibition (PPI), phosphatidylinositol-3-kinase (PI3K), auditory cortex (AC), auditory brainstem response (ABR), medial geniculate body (MGB), inferior colliculus (IC), ventral nucleus of the lateral lemniscus (VNLL), superior paraolivary nucleus (SPON), medial nucleus of the trapezoid body (MNTB), valproic acid (VPA).

Model	Gene	Mouse locus	Human locus	Protein	Protein function	Main findings
***Shank3*-KO**	*Shank3*	15; 15 E3	22q13.3	SHANK3 (SH3 and multiple ankyrin repeat domains protein 3)	Scaffold protein important for organization of excitatory synapses and integrity of the post-synaptic density (Monteiro and Feng, 2017).	Increased ASR, decreased PPI, increased ability for pitch discrimination (Zhou et al., 2016; Rendall et al., 2019). Weaker cortical responses to sounds, temporal processing is impaired when sounds are presented at high rate, resembling speech rate (Engineer et al., 2018).
***Pten* conditional KO**	*Pten*	19 C1; 19 28.14 cM	10q23.31	PTEN (phosphatase and tensin homolog)	Negative regulator of the PI3K/AKT signaling, important for cell growth, proliferation and survival, and axonal growth (Zhou and Parada, 2012; Sarn et al., 2021).	Strengthened synaptic connectivity: stronger callosal inputs to the AC from contralateral AC and thalamus (Xiong et al., 2012).
***Cntnap2*-KO**	*Cntnap2*	6; 6 B2.2- B2.3	7q35-q36.1	CNTNAP2 (Contactin associated protein 2)	Maintenance of normal action potential propagation through tethering of potassium channels in myelinated axons (Truong et al., 2015).	Increased pitch discrimination, but impaired temporal processing of sounds. Altered ABRs (peak amplitudes and latencies), histological alterations in MGB (Truong et al., 2015; Scott et al., 2018).
***α7-nAChR*-KO**	*Chrna7*	7 C; 7 34.47 cM	15q13.3	α7-AChR (α7-nicotinic acetylcholine receptor)	Ligand-gated cationic channel that interacts with acetylcholine and mediates synaptic transmission (Schaaf, 2014; De Jaco et al., 2016).	Increased latency of ABR peak IV, atypical spectral and temporal response of IC neurons. Degraded spike timing in VNLL and SPON (Felix et al., 2019).
***Fmr1*-KO**	*Fmr1*	X A7.1; X 34.83 cM	Xq27.3	FMRP (Fragile X Mental Retardation Protein)	Actin remodeling, regulation of cytoskeleton-related gene expression (Braun and Segal, 2000).	Behavioral evidence of deficits in sound processing and hyperacusis (Reinhard et al., 2019; Auerbach et al., 2021). Altered cortical responses to different types of stimuli (Rotschafer and Razak, 2013), higher ABR thresholds (Rotschafer et al., 2015) and lack of tonotopicity in MNTB (Strumbos et al., 2010; Ruby et al., 2015), IC (Nguyen et al., 2020) and AC (Rotschafer and Razak, 2013).
***Mecp2*-TG**	*Mecp2*	X A7.3; X 37.63 cM	2p16.3	MECP2 (methyl-CpG binding protein 2)	Mainly expressed in the brain. Gene expression regulator, important for prenatal neurogenesis and synaptic development (Brand et al., 2021).	Normal ABR and cortical tonotopy. Increased thresholds for tone-evoked cortical responses, heightened and delayed cortical responses to noise (Zhou et al., 2019a).
**VPA**	*N/A*	*N/A*	*N/A*	*N/A*	*N/A*	Reduced USVs throughout life, normal ASR but reduced PPI suggesting sensory-motor gating impairments (Gandal et al., 2010). Cortical alterations on LFPs, temporal and spectral processing, and tonotopicity (Engineer et al., 2014a; Anomal et al., 2015). Auditory brainstem overactivation upon sound stimulation (Dubiel and Kulesza, 2016). Impaired connectivity between the brainstem and the midbrain, which is one of the most susceptible regions to VPA exposure (Zimmerman et al., 2020).
**Thalidomide**	*N/A*	*N/A*	*N/A*	*N/A*	*N/A*	Impairments in MNTB: diminished in size, fewer projections, expanded responsive area to sound stimulation (Ida-Eto et al., 2017; Tsugiyama et al., 2020).
